# Impact of a National Tobacco Education Campaign on Weekly Numbers of Quitline Calls and Website Visitors — United States, March 4–June 23, 2013

**Published:** 2013-09-20

**Authors:** Mary Anne Bright, Kevin Davis, Stephen Babb, Rebecca Bunnell, Robert Rodes, Robert Alexander, Caryn Coln, Lei Zhang, Diane Beistle, Jane Mitchko, Timothy McAfee

**Affiliations:** National Cancer Institute, Bethesda, Maryland; RTI International, Research Triangle Park, North Carolina; Office on Smoking and Health, National Center for Chronic Disease Prevention and Health Promotion, CDC

During March 4–June 23, 2013, CDC conducted its second annual national paid-media tobacco education campaign encouraging adult smokers to quit. These campaigns, called Tips from Former Smokers (Tips), feature true stories of former smokers living with serious smoking-related diseases. To assess the immediate impact of the 2013 Tips campaign, CDC analyzed the weekly numbers of calls to the national telephone quitline portal (1-800-QUIT-NOW) and the weekly numbers of unique visitors to the Tips website ( http://www.cdc.gov/tips )* during the 16-week campaign and during the 4 weeks before and after the campaign. During the campaign, the average weekly numbers of calls and website visitors increased by 75% and almost 38-fold, respectively, compared with the 4 weeks before the campaign, and quickly decreased almost to pre-campaign levels once the campaign ended. This suggests that the campaign led to 151,536 additional quitline calls and nearly 2.8 million additional unique Tips website visitors above pre-campaign levels. During the first 12 weeks of the campaign,† when the national television ads were on and off air on alternate weeks, average weekly call volume fell by 38% during the 6 weeks when the national television ads were off air compared with the 6 weeks when these ads were running. These results suggest that emotionally evocative tobacco education media campaigns featuring graphic images of the health effects of smoking can increase quitline calls and website visits and that these campaigns’ effects decrease rapidly once they are discontinued.

The 2013 campaign included advertising on television, online (Internet and mobile), radio, print, billboards, buses and bus stop shelters, and social media. The campaign’s television component included national ads in all 210 U.S. designated market areas (DMAs) and additional local ads in 67 of these DMAs, which were selected randomly.§ To extend the campaign’s length, the national television ads were placed using a “pulsing” strategy, which involved airing them on a 1-week-on, 1-week-off basis through the first 12 weeks of the campaign, while the local television ads ran continuously throughout the campaign. The campaign’s online component consisted of national ads only and ran continuously throughout the campaign.

The campaign’s television ads included one of three calls to action: an invitation to call 1-800-QUIT-NOW (66%), an invitation to visit the Tips website (28%), and the message “Talk with your doctor (6%).”¶ During the campaign’s first 12 weeks, all online ads included the Tips website; during its last 4 weeks, these ads included a mix of the Tips website, 1-800-QUIT-NOW, and “Talk with your doctor.” In addition to featuring Tips ads, which provide motivation to quit smoking, the Tips website syndicated extensive information on how to quit from the National Cancer Institute’s (NCI’s) cessation website (http://www.smokefree.gov).

This analysis used 1-800-QUIT-NOW call volume data collected by NCI from the national portal and data on unique visitors to the Tips campaign website collected by CDC using Adobe SiteCatalyst and Google Analytics. For the purposes of this report, unique visitors are defined as persons who visited the Tips website one or more times in a given week.

A total of 352,848 calls to 1-800-QUIT-NOW occurred during the 2013 campaign, for a weekly average of 22,053 calls (Figure 1).** This represents 151,536 additional calls (a 75% increase) above what would have been expected had call volume continued at the level observed during the 4 weeks before the campaign (February 4–March 3), when the weekly average was 12,582 calls. The average weekly call volume of 13,044 calls during the 4 weeks after the campaign (June 24–July 21) was 41% lower than the weekly average observed during the campaign.†† Both the increase in call volume from the pre-campaign weeks to the campaign weeks and the decrease in call volume from the campaign weeks to the post-campaign weeks were statistically significant (p<0.05), whereas no significant difference in call volume was found between the pre-campaign and post-campaign weeks (p=0.60).§§

During the campaign’s first 12 weeks, average weekly call volume was 38% lower during the 6 weeks when the national television ads were off-air compared with the 6 weeks when these ads were running (16,500 versus 26,571). A separate analysis found that during off-air weeks, call volume in DMAs without local ads fell nearly to the level of the 4 pre-campaign weeks.

Nearly 2.9 million (2,868,059) unique visitors accessed the Tips campaign website during the 2013 Tips campaign, for a weekly average of 179,254 unique visitors (Figure 2). This represents almost 2.8 million (2,792,475) additional unique visitors (a nearly 38-fold increase) above what would have been expected had website traffic continued at the level observed during the 4 weeks before the campaign, when the weekly average was 4,724 visitors. The weekly average of 7,575 website visitors during the 4 post-campaign weeks was 96% lower than the weekly average observed during the campaign. The cessation sections of the English and Spanish Tips websites received almost half a million page views during the campaign, suggesting that many visitors to the Tips website were actively seeking information on how to quit smoking. Although the weekly number of website visitors varied during the course of the campaign, this variation did not clearly follow the pattern of the national television ad pulsing.

## Editorial Note

Emotionally evocative tobacco education media campaigns featuring graphic images of smoking-related diseases are effective in motivating smokers to quit (1–5). Telephone quitlines increase quit rates (6). The 1-800-QUIT-NOW portal, operated by NCI, seamlessly routes callers to their state quitlines based on area code. CDC funds state quitlines as part of its support for comprehensive state tobacco control programs. Web-based cessation interventions are promising (6), but the available evidence is insufficient to fully assess their effectiveness (7).

This analysis shows that the number of weekly calls to 1-800-QUIT-NOW increased as soon as the Tips campaign began, decreased when the national television ads were off-air, increased each time these ads returned to the air, and decreased when the campaign ended. Each of these increases and decreases was rapid and substantial. These findings reinforce previous evidence that media campaigns can motivate smokers to try to quit and to seek information on quitting (1–5), while also offering additional evidence that these campaigns’ effects fade quickly once they end (2,3,8). These findings underscore the public health importance of sustaining campaigns over time, and suggest that the Tips campaign might have generated even more quitline calls if the national television ads had run continuously and had appeared over a longer period. If the national television ads had been aired continuously without pulsing over the entire 16 weeks of the campaign, assuming the average weekly call volume observed during the first 6 “on” weeks was sustained, about 425,000 calls would have occurred during the campaign (i.e., about 72,000 more calls than actually occurred).

As with 1-800-QUIT-NOW calls, the weekly number of unique visitors to the Tips campaign website increased sharply immediately after the campaign began and decreased sharply once it ended. However, although the number of weekly website visitors varied during the campaign, the pulsing pattern was far less evident than it was for quitline calls. This suggests that the national online ads, which ran continuously throughout the campaign, were a greater driver of website traffic than the pulsed national television ads. This is plausible, because online ads have a built-in advantage in directing traffic to a website; online ad viewers need only click on an ad to visit the website. In addition, only about 28% of the television ads included the website. During the weeks of April 22–28 and June 17–23, the amount of online advertising increased sharply, corresponding with substantial increases in unique website visitors, indicating that website traffic is responsive to changes in the online ad exposure dose. The fact that there were almost 3 million visitors to the Tips website during the 2013 campaign suggests that online ads hold promise for motivating smokers to engage in cessation information-seeking activity.

Although substantial increases occurred in the numbers of 1-800-QUIT-NOW calls and Tips website visitors during the 2013 Tips campaign, total numbers of calls and website visitors during the 2013 campaign are somewhat smaller than during the 2012 Tips campaign (9).¶¶ This likely reflects at least two factors. First, the national television and online ad purchases were lower in 2013 than in 2012. Second, national television ads including 1-800-QUIT-NOW ran for only 9 weeks in 2013, compared with 12 weeks in 2012.

What is already known on this topic?

The number of weekly calls to a national quitline portal and the number of weekly unique visitors to a cessation website increased substantially during the 2012 Tips from Former Smokers national tobacco education media campaign (Tips campaign) compared with the same period in 2011.

What is added by this report?

The number of weekly calls to the national quitline portal and the number of weekly unique visitors to the Tips campaign website increased substantially during the 2013 Tips campaign, compared with the 4 weeks preceding the campaign. These numbers decreased rapidly once the campaign ended. Calls also decreased sharply during campaign weeks when the national television ads were off the air. Increases in quitline call volume appeared to be driven primarily by television ads, whereas increases in traffic to the Tips website appeared to be driven primarily by online ads.

What are the implications for public health practice?

Tobacco education media campaigns featuring true personal stories from former smokers with serious smoking-related diseases and graphic images can substantially increase calls to quitlines and visitors to campaign websites, which would be expected to result in increases in quit attempts and successful quit attempts. Quitline calls and website visitors fall sharply when campaigns are discontinued. Media campaigns such as the Tips campaign might have an even greater impact if they were sustained at a high intensity for a longer time.

The findings in this report are subject to at least five limitations. First, this analysis was a natural time series analysis and did not control for other factors that could have contributed to the increases in weekly call volume and website visitors observed during the 2013 Tips campaign. However, the observed impact of the campaign’s pulsing strategy on call volume, as well as the sharp increase and decrease in calls and website visitors observed immediately after the campaign began and ended, point to a causal relationship. Secondly, NCI data on calls to 1-800-QUIT-NOW reflect the number of call attempts, not the number of unique callers, completed calls, or callers receiving services. The NCI data also do not capture calls to other state quitline numbers besides 1-800-QUIT-NOW. Third, adding the weekly numbers of Tips website unique visitors to calculate total unique visitors during the campaign likely somewhat overestimates this total because the same person could be counted several times if they visited the Tips website repeatedly over several different weeks. Fourth, the number of Tips website visitors included both web and mobile visitors, so some persons who visited the website through both these channels could have been counted twice, resulting in an overestimate. Finally, 1-800-QUIT-NOW calls and visitors to the Tips website are preliminary indicators of quit attempts and successful cessation, outcomes which future evaluations of the 2013 Tips campaign will examine. However, the 2012 Tips campaign was associated with increases in call volume, website visitors, quit attempts, and successful cessation, suggesting that the first two indicators predict the latter two outcomes (9,10).

Calls to quitlines and traffic to the campaign website increased sharply when the Tips campaign was on the air, suggesting that this campaign likely motivated many smokers to try to quit. Quitline call volume appeared to be driven primarily by television ads, whereas traffic to the Tips website appeared to be driven primarily by online ads. Both quitline calls and website traffic fell rapidly when the campaign was discontinued. These findings speak to the effectiveness of emotionally evocative media campaigns featuring graphic images in increasing interest in quitting smoking, and highlight the even greater impact these campaigns might have if they were sustained at a high intensity for a longer time. If the national television ads that aired during “on” weeks of the 2013 Tips campaign were run throughout the year at this level and produced the same response, this would translate into almost 1.4 million calls to 1-800-QUIT-NOW in 2013, which is 500,000 more calls than the portal received in 2012 and by far the most calls that it has received in any year since its inception.

## Figures and Tables

**FIGURE 1:**
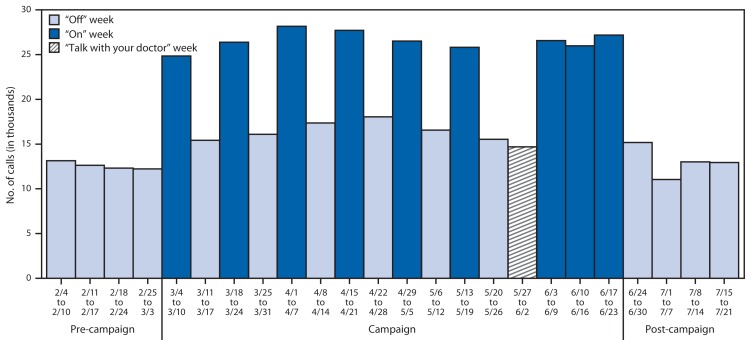
Number of weekly telephone calls made to 1-800-QUIT-NOW before, during, and after CDC’s 2013 Tips from Former Smokers campaign — United States, February 4–July 21, 2013* * For the week of May 27–June 2, the national television ads were running, but most of these ads featured the message “You can quit – talk with your doctor for help.” For the weeks of June 3–9, June 10–16, and June 17–23, a substantial proportion of online ads were tagged with 1-800-QUIT-NOW. For the week of June 24–30, some television stations continued to run ads for a short period after the campaign ended.

**FIGURE 2:**
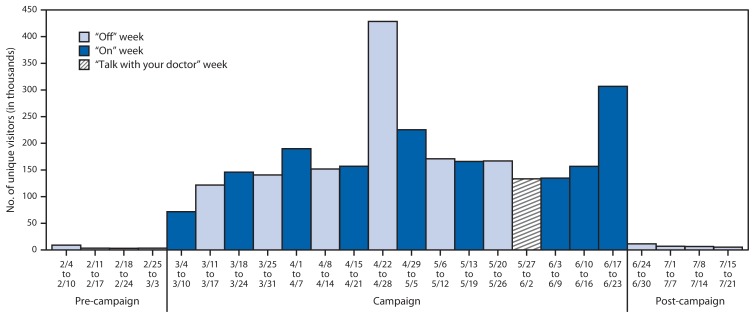
Number of weekly unique visitors to campaign websites before, during, and after CDC’s 2013 Tips from Former Smokers campaign — February 4–July 21, 2013* * For the week of April 22–28, YouTube mistakenly ran substantially more online ads than were purchased. For the week of May 27–June 2, the national television ads were running, but most of these ads featured the message “You can quit – talk with your doctor for help.” For the weeks of June 3–9, June 10–16, and June 17–23, a substantial proportion of online ads were tagged with 1-800-QUIT-NOW rather than the website address. For the week of June 17–23, online ad impressions were run at an especially high level. For the week of June 24–30, some ad servers continued to run online ads for a short period after the campaign ended.

## Footnotes

* In addition to unique visitors to http://www.cdc.gov/tips (English), the numbers reported include unique visitors to the associated resources http://www.cdc.gov/consejos (Spanish), http://m.cdc.gov/tips (English, mobile platform), and http://m.cdc.gov/consejos (Spanish, mobile platform).

† This part of the analysis focuses on the first 12 weeks of the campaign because the calls to action featured on the television and online ads and the pulsing schedule changed substantially during the last 4 weeks of the campaign.

§ Because of resource constraints, the 20 largest DMAs in the United States were excluded from the randomization process and received the national television ads only.

¶ During the 9th week of the campaign (May 27–June 2), most television ads included this message. The full message was “You can quit – talk with your doctor for help.”

** The campaign call totals reported in this analysis do not include almost 150,000 calls that were generated by mobile “click-to-call” promotions beginning April 1. These calls were excluded from the analysis because reports from several state quitlines suggested that few, if any, of these callers remained on the line to receive services.

†† Some television stations continued to run ads for a short period after the campaign ended.

§§ The statistical test applied was a t-test comparison of the difference in means.

¶¶ This comparison of website traffic in the 2012 and 2013 Tips campaigns takes into account that the 2012 ads featured one of two websites (CDC’s Tips website and NCI’s smokefree.gov website), whereas the 2013 ads included a single website (the Tips website). Accordingly, 2,868,059 visitors to the Tips website during the 2013 campaign are being compared with the combination of 629,898 unique visitors to the smokefree.gov website and the most comparable available indicator of 2,750,420 visits to the Tips website during the 2012 campaign. (The latter figure is for unique visits, not unique visitors. CDC was unable to track unique visitors to the Tips campaign website in 2012 because of agency regulations in effect at that time. The number of unique visitors to a website typically is somewhat smaller than the number of unique visits.)
